# Supplementary material for: Single-molecule nanophotonic resolution of binding dynamics from apo to fully liganded for a cyclic nucleotide-gated ion channel in cell-derived vesicles
Source: bioRxiv. 2026 May 27:2026.05.24.727213. Preprint. [Version 1] doi: 10.64898/2026.05.24.727213 (PMC13232105; doi:10.64898/2026.05.24.727213)
Supplement: Supplementary file 1 [file NIHPP2026.05.24.727213v1-supplement-1.pdf]

bioRxiv preprint doi: https://doi.org/10.64898/2026.05.24.727213; this version posted May 27, 2026. The copyright holder for this preprint (which was not certified by peer review) is the author/funder, who has granted bioRxiv a license to display the preprint in perpetuity. It is made available under a CC-BY 4.0 International license.

Supplementary Information for "Single-molecule nanophotonic resolution of binding dynamics from apo to fully liganded for a cyclic nucleotide-gated ion channel in cell-derived vesicles"

Tapas Haldar[1], Draegan Watson[1], Cecilia M. Borghese[1], Zaid Ahmed[1], Perla A. Peña Palomino[1], Susanne Ressl[1], Audrey C. Brumback[1-3], and Marcel P. Goldschen-Ohm[1]*

[1]Department of Neuroscience, University of Texas at Austin
[2]Department of Neurology, Dell Medical School at the University of Texas at Austin
[3]Department of Pediatrics, Dell Medical School at the University of Texas at Austin
*Correspondence to marcel.goldschen-ohm@austin.utexas.edu

bioRxiv preprint doi: https://doi.org/10.64898/2026.05.24.727213; this version posted May 27, 2026. The copyright holder for this preprint (which was not certified by peer review) is the author/funder, who has granted bioRxiv a license to display the preprint in perpetuity. It is made available under a CC-BY 4.0 International license.

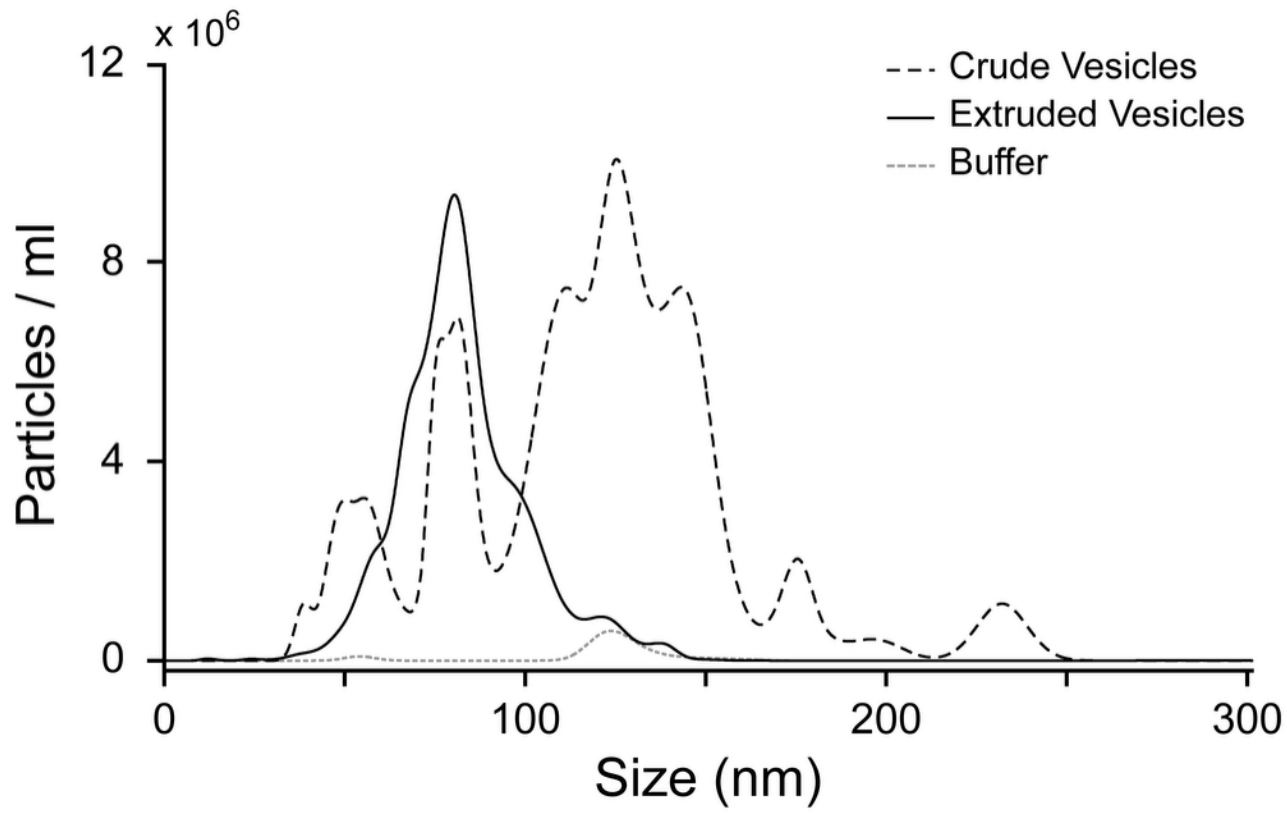

Figure S1. Extruded vesicle size distribution.

Size distributions for non-extruded (crude, dashed line) or extruded (solid line) cell-derived vesicles. Control is for PBS buffer alone which had minimal particulate contaminants. Vesicle samples contained the TAX-4 fusion protein as described in the main text. Size distributions were obtained by nanoparticle tracking analysis (NTA) using a NanoSight NS500 (NanoSight, Amesbury, United Kingdom) equipped with a 488 nm laser.

bioRxiv preprint doi: https://doi.org/10.64898/2026.05.24.727213; this version posted May 27, 2026. The copyright holder for this preprint (which was not certified by peer review) is the author/funder, who has granted bioRxiv a license to display the preprint in perpetuity. It is made available under a CC-BY 4.0 International license.

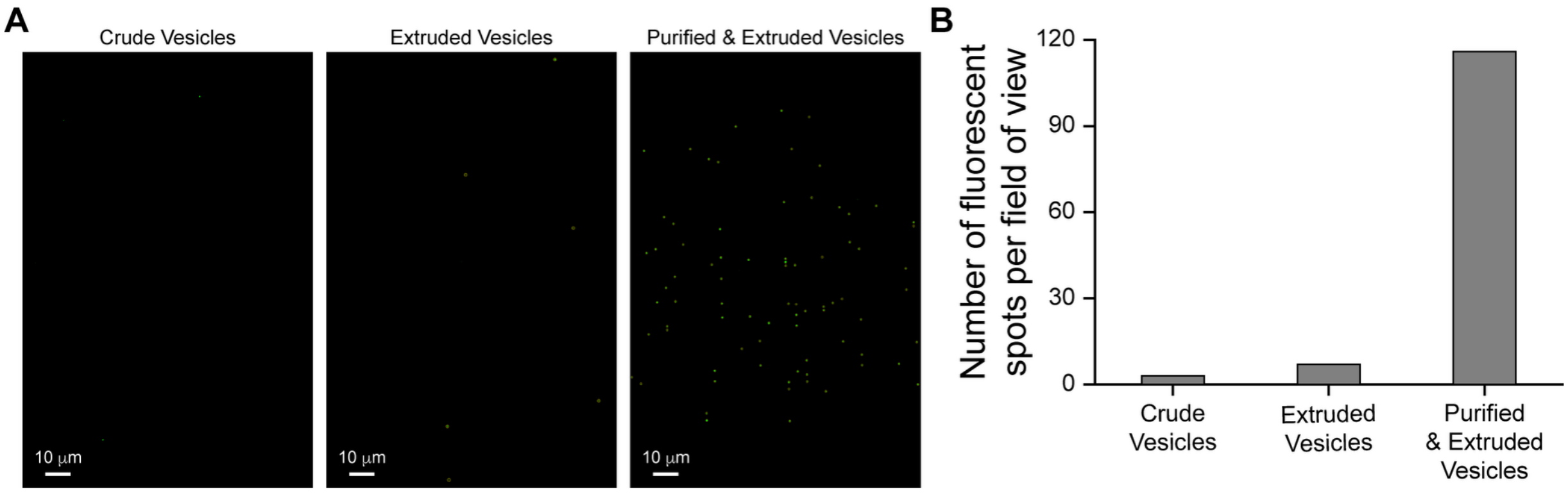

Figure S2. Loading TAX-4 containing cell-derived vesicles into ZMW arrays.

(A) Fluorescence images of 150 nm ZMW arrays after loading with either crude or extruded vesicles containing the fusion protein eGFP–TAX-4 (left, middle), or purified and extruded inside-out cell-derived vesicles containing the fusion protein ALFA–mSG–TAX-4. Fluorescence is from mSG (right). **(B)** Each fluorescent spot is indicative of a ZMW well containing a vesicle with a TAX-4 fusion protein. Relatively efficient loading was only observed for samples that were both purified and extruded.

bioRxiv preprint doi: https://doi.org/10.64898/2026.05.24.727213; this version posted May 27, 2026. The copyright holder for this preprint (which was not certified by peer review) is the author/funder, who has granted bioRxiv a license to display the preprint in perpetuity. It is made available under a CC-BY 4.0 International license.

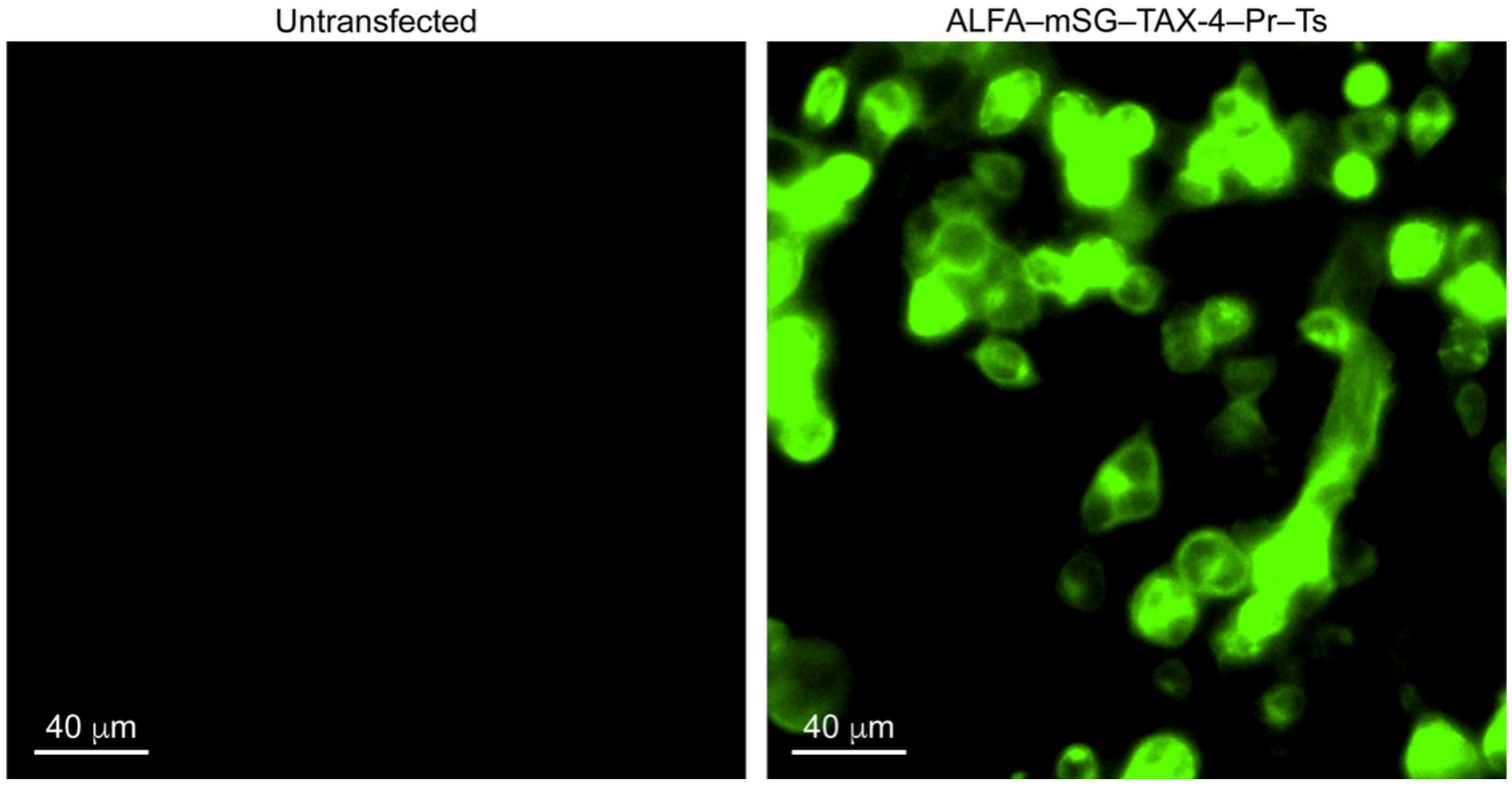

Figure S3. Expression of the fusion protein ALFA–mSG–TAX-4–PS–TS in HEK293T cells.

Fluorescence emission from mStayGold (mSG) excited at 488 nm in HEK-293T cells either untransfected (left) or transfected with the fusion protein ALFA–mSG–TAX-4–PS–TS (right).

bioRxiv preprint doi: https://doi.org/10.64898/2026.05.24.727213; this version posted May 27, 2026. The copyright holder for this preprint (which was not certified by peer review) is the author/funder, who has granted bioRxiv a license to display the preprint in perpetuity. It is made available under a CC-BY 4.0 International license.

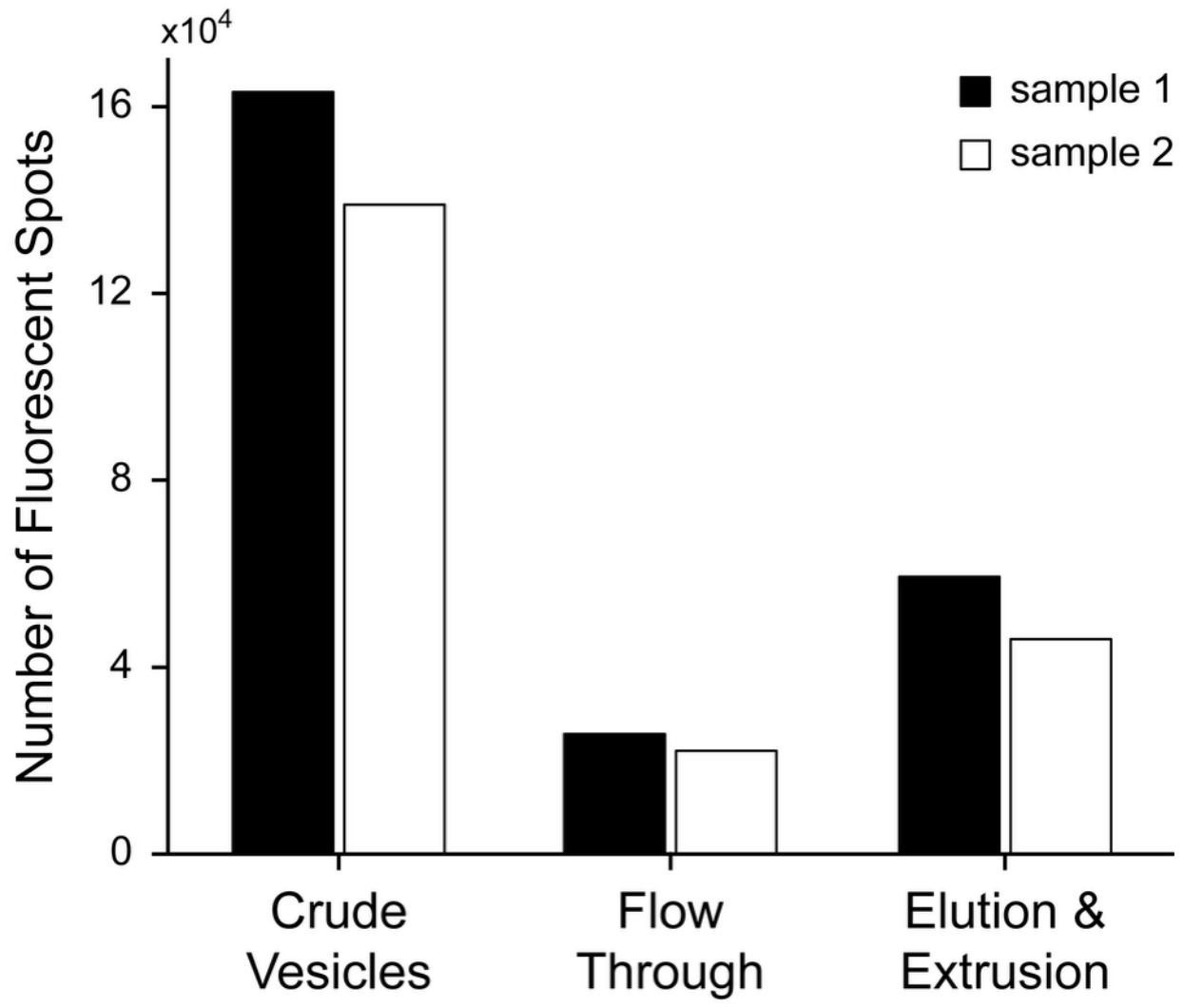

Figure S4. Purification of ALFA–mSG–TAX-4 in cell-derived vesicles.

Fluorescence from mSG was used to quantify the amount of vesicles containing ALFA–mSG–TAX-4 during the purification process. We started with a 320 µl suspension of crude vesicles from the initial vesicle preparation which is comprised of vesicles both with and without the TAX-4 fusion protein in both inside-out and outside-out configurations. A 10 µl aliquot of the crude suspension was placed on a glass coverslip and imaged with TIRF illumination at 488 nm. The total number of fluorescent spots (e.g., vesicles) in a single field of view (F) was recorded and scaled by 32 to estimate the same measure for the entire sample. The remaining 310 µl was taken forward for purification and incubated with Step-Tactin coated magnetic beads. The beads were then washed with buffer to remove unbound vesicles and the flow through collected. A 50 µl aliquot of the flow through (300 µl total) was imaged as described for the crude vesicles and F was scaled by 6 to estimate the same measure for the entire flow through. Finally, the vesicles remaining bound to the beads were eluted by enzymatic cleavage at the PreScission site and the elute was collected and extruded in a final volume of 750 µl. A 50 µl aliquot of the extruded sample was imaged as described for the crude vesicles and F was scaled by 15 to estimate the same measure for the entire eluted and extruded sample. Scaled values of F at each step are shown for two separate samples on different days. Most of the TAX-4 containing vesicles remained bound to the beads, with only a small fraction ending up in the flow through. Roughly half of the bead-associated vesicles were eluted in the purified sample. Purification efficiency was similar for both samples.

bioRxiv preprint doi: https://doi.org/10.64898/2026.05.24.727213; this version posted May 27, 2026. The copyright holder for this preprint (which was not certified by peer review) is the author/funder, who has granted bioRxiv a license to display the preprint in perpetuity. It is made available under a CC-BY 4.0 International license.

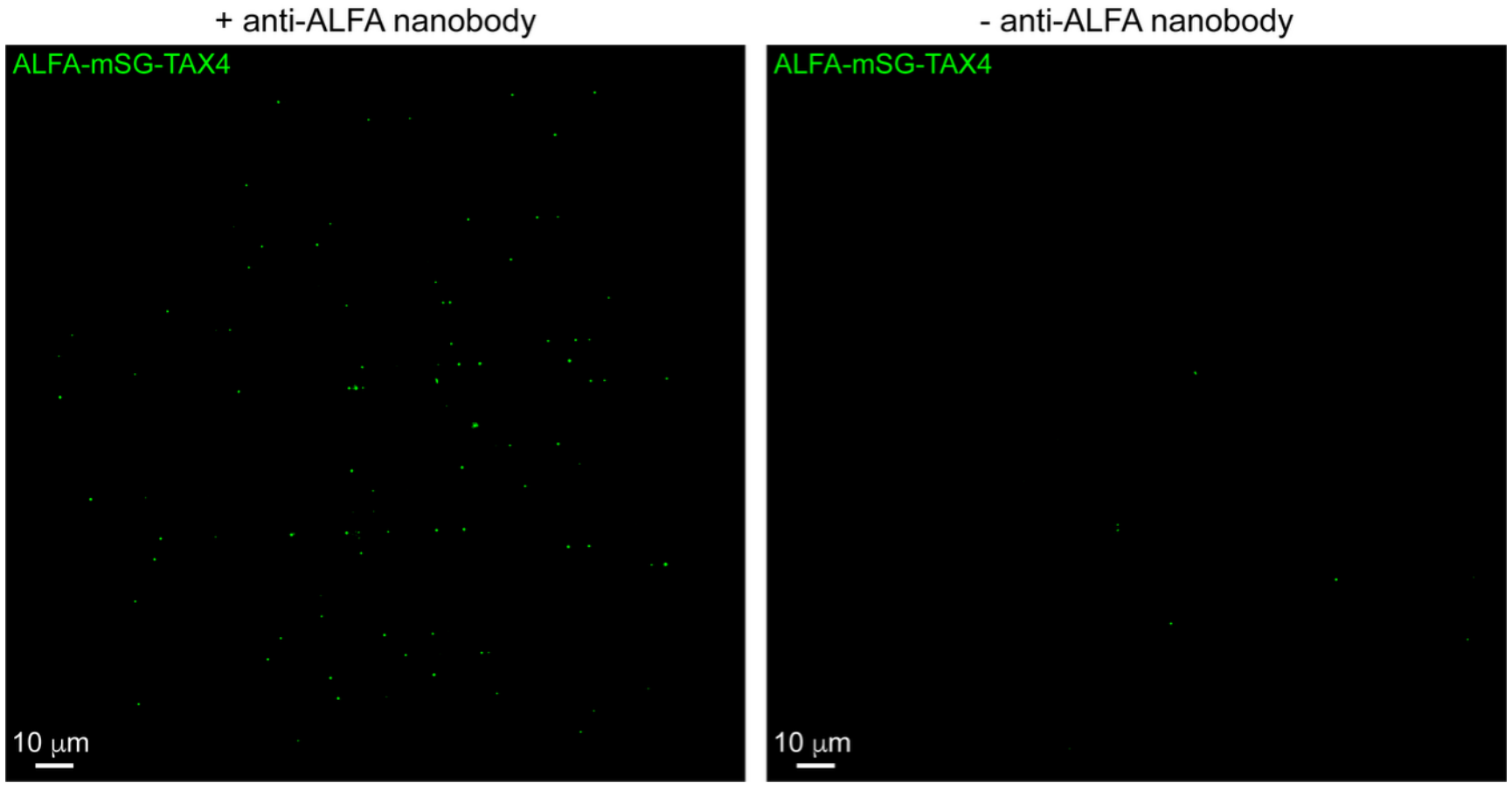

Figure S5. Specific immobilization of ALFA–mSG–TAX-4 containing vesicles.

Fluorescence images of a ZMW array with (left) or without (right) anti-ALFA nanobodies on the bottom of the nano-wells after loading with cell-derived vesicles containing ALFA–mSG–TAX-4. Fluorescence from mSG is rarely observed in the absence of the nanobody, suggesting that mSG spots largely reflect specific immobilization via anti-ALFA nanobodies as opposed to non-specific adsorption of vesicles.

bioRxiv preprint doi: https://doi.org/10.64898/2026.05.24.727213; this version posted May 27, 2026. The copyright holder for this preprint (which was not certified by peer review) is the author/funder, who has granted bioRxiv a license to display the preprint in perpetuity. It is made available under a CC-BY 4.0 International license.

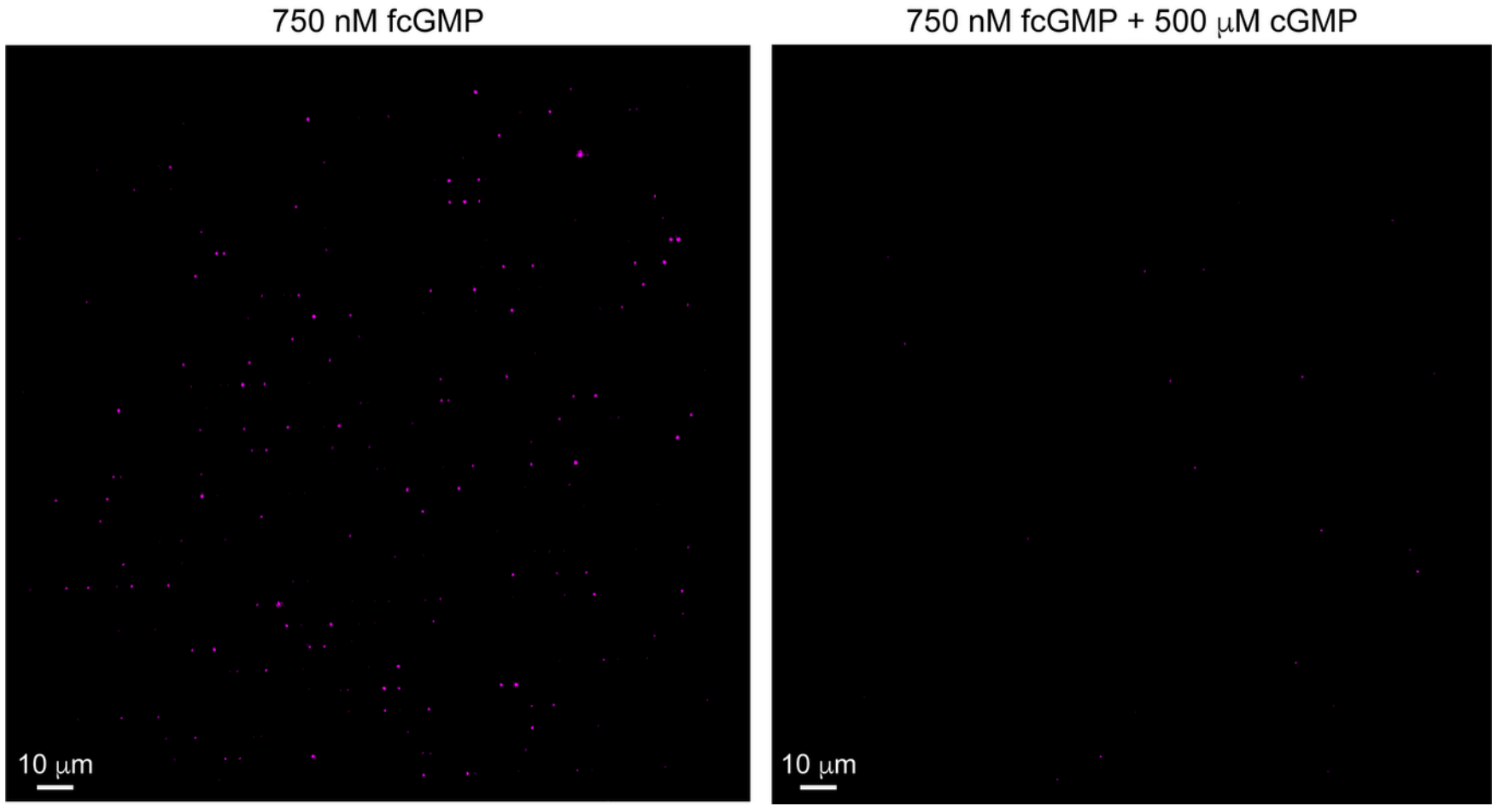

Figure S6. Specific binding of fcGMP to TAX-4 channels in cell-derived vesicles within ZMWs.

Time-averaged fluorescence images for fcGMP binding. Both images are for the same field of view. Robust binding in 750 nm fcGMP (left) is largely abolished by competition with an excess of non-fluorescent cGMP (right).

bioRxiv preprint doi: https://doi.org/10.64898/2026.05.24.727213; this version posted May 27, 2026. The copyright holder for this preprint (which was not certified by peer review) is the author/funder, who has granted bioRxiv a license to display the preprint in perpetuity. It is made available under a CC-BY 4.0 International license.

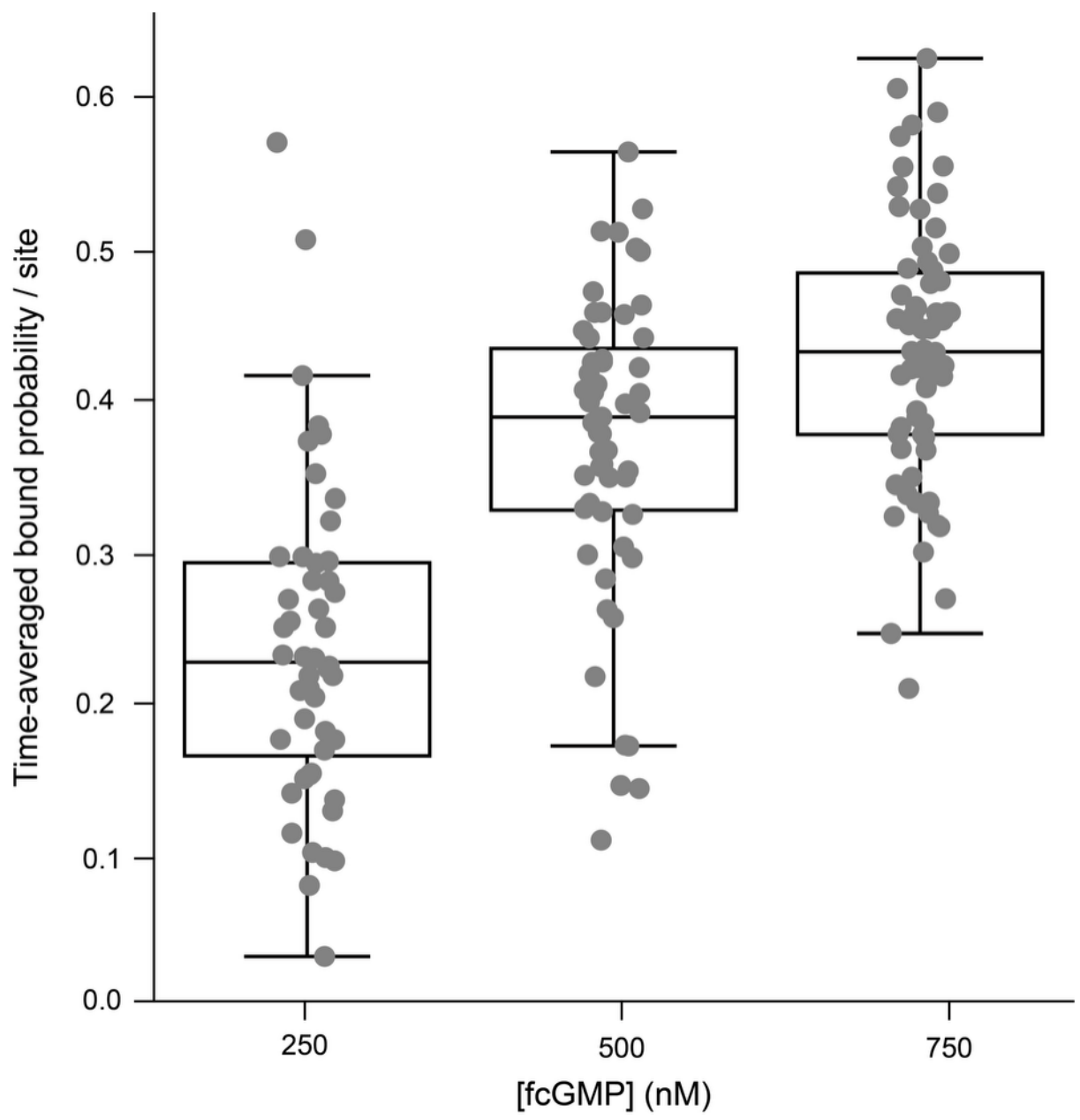

Figure S7. Probability of binding site occupation as a function of fcGMP concentration

Each solid circle is the time-averaged bound probability per site for an individual TAX-4 channel. Box plots indicate the median and quartiles. The binding probability increases with increasing fcGMP ligand concentration, demonstrating a concentration-dependent interaction between TAX-4 containing nanovesicles and the fcGMP ligand.

bioRxiv preprint doi: https://doi.org/10.64898/2026.05.24.727213; this version posted May 27, 2026. The copyright holder for this preprint (which was not certified by peer review) is the author/funder, who has granted bioRxiv a license to display the preprint in perpetuity. It is made available under a CC-BY 4.0 International license.

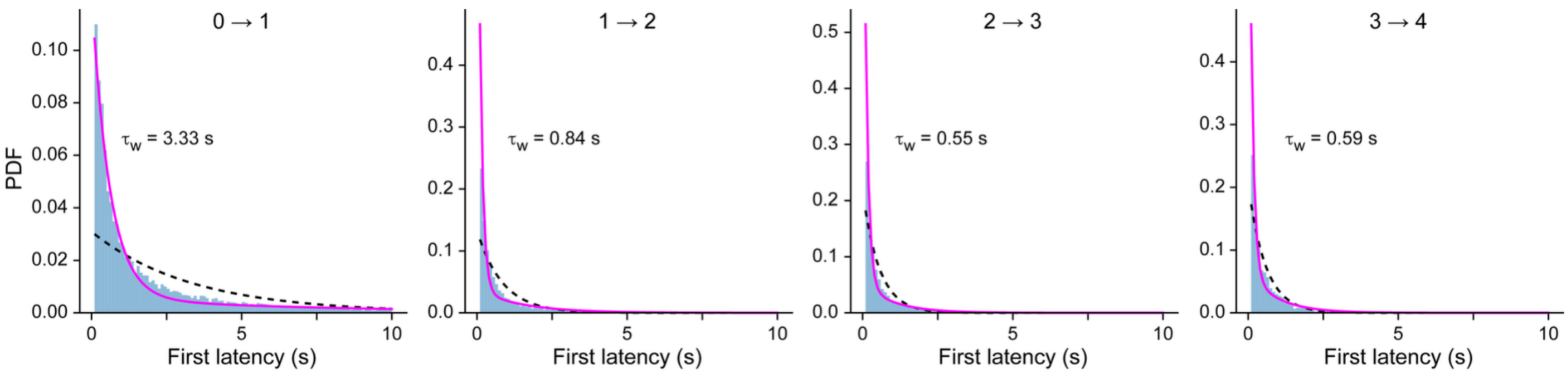

Figure S8. Step-wise binding first latencies.

Distributions of first latencies to fcGMP binding for stepwise binding at four binding sites in TAX-4 channels. Mono-exponential (black dashed line) and bi-exponential (solid magenta line) maximum likelihood fits are shown, and the weighted time (τ_w) constant from bi-exponential fits is shown.

bioRxiv preprint doi: https://doi.org/10.64898/2026.05.24.727213; this version posted May 27, 2026. The copyright holder for this preprint (which was not certified by peer review) is the author/funder, who has granted bioRxiv a license to display the preprint in perpetuity. It is made available under a CC-BY 4.0 International license.

	Binding (μM^{-1}s^{-1})				Unbinding (s^{-1})			
**Model**	$k_{0\to1}$	$k_{1\to2}$	$k_{2\to3}$	$k_{3\to4}$	$k_{4\to3}$	$k_{3\to2}$	$k_{2\to1}$	$k_{1\to0}$
B$_i$	$4k_{3\to4}$	$3k_{3\to4}$	$2k_{3\to4}$	0.7 ± 0.2	$4k_{1\to0}$	$3k_{1\to0}$	$2k_{1\to0}$	0.7 ± 0.2
B$_c$	2.3 ± 0.4	2.2 ± 0.6	1.7 ± 0.6	1.0 ± 0.4	1.7 ± 0.6	1.7 ± 0.6	1.2 ± 0.3	0.9 ± 0.2
G$_i$	$4k_{3\to4}$	$3k_{3\to4}$	$2k_{3\to4}$	0.7 ± 0.2	$4k_{1\to0}$	$3k_{1\to0}$	$2k_{1\to0}$	2.0 ± 0.4
G$_c$	2.4 ± 0.5	2.2 ± 0.7	1.7 ± 0.6	1.0 ± 0.4	6.4 ± 1.1	5.7 ± 1.3	3.8 ± 0.7	2.0 ± 0.4
S$_i$	$4k_{3\to4}$	$3k_{3\to4}$	$2k_{3\to4}$	0.7 ± 0.2	$4k_{1\to0}$	$3k_{1\to0}$	$2k_{1\to0}$	1.9 ± 0.4
S$_c$	2.5 ± 0.5	2.3 ± 0.7	1.8 ± 0.7	1.0 ± 0.4	3.9 ± 1.4	4.2 ± 1.3	3.5 ± 0.9	2.6 ± 0.5

	Isomerization (s^{-1})				
**Model**	k_i	k_r	α	*dof*	**ΔBIC**
B$_i$	-	-	-	2	29,212
B$_c$	-	-	-	8	25,115
G$_i$	0.1 ± 0.02	0.3 ± 0.01	0.16 ± 0.01	5	4,339
G$_c$	0.1 ± 0.02	0.2 ± 0.02	0.17 ± 0.02	11	3,161
S$_i$	0.4 ± 0.03	0.3 ± 0.03	-	4	3,983
S$_c$	0.4 ± 0.05	0.2 ± 0.03	-	10	0

Table S1. Model parameters.

Parameters for kinetic models as shown in Fig. 4 of the main text. Units are μM^{-1}s^{-1} for binding rates and s^{-1} for unbinding and isomerization rates, and α is unitless. Parameters are from a global fit to a combined dataset including data at 10, 30, 60, 100, 200, 250, 500, and 750 nM fcGMP. Data at 250-750 nM fcGMP are from this work, and data from 10-200 nM fcGMP are from our previous publication (Patel et al. 2021). See methods in the main text. Error is the standard deviation for separate fits to three unique stratified folds of the combined dataset with approximatley equal representation at each concentration. Constraints are indicated by shaded cells. Degrees of freedom (*dof*) is the number of free parameters in each model and ΔBIC are the models' relative Bayesian Information Criterion scores (lower is better).
